# Cultural Competence in Refugee Service Settings: A Scoping Review

**DOI:** 10.1089/heq.2020.0094

**Published:** 2021-03-16

**Authors:** Ling San Lau, Graeme Rodgers

**Affiliations:** ^1^ Program on Forced Migration and Health, Heilbrunn Department of Population and Family Health, Mailman School of Public Health, Columbia University, New York, New York, USA.; ^2^ International Rescue Committee, New York, New York, USA.

**Keywords:** refugee, asylum seeker, cultural competence

## Abstract

**Purpose:** Refugees and asylum seekers have unique and complex needs related to their experiences of forced displacement and resettlement. Cultural competence is widely recognized as important for the provision of effective and equitable services for refugee populations. However, the delivery of culturally appropriate services—including health care and social services—is often complicated by unclear definitions and operationalization of cultural competence. Further, the unique needs and priorities of people from refugee backgrounds are under-addressed in the cultural competence literature. This scoping review seeks to synthesize the peer-reviewed literature examining cultural competence in refugee service settings.

**Methods:** A systematic search of four databases (EBSCO, Proquest, Scopus and Google Scholar) identified 26 relevant peer-reviewed studies for analysis.

**Results:** A range of approaches to cultural competence were identified at the level of individual providers and organizations.

**Conclusion:** We identified a need for greater refugee participation and perspectives in the practice of cultural competence, increased conceptual clarity and greater recognition of structural barriers. We call for further rigorous research that critically examines the concept of cultural competence and its meaning and relevance to refugee populations.

## Introduction

Refugees and asylum seekers include persons who have fled their countries due to war or persecution. In common with other marginalized populations, they experience challenges accessing services that address their individual and cultural needs. Cultural competence is widely recognized as a critical component of effective and equitable service delivery^1^ and has been proposed to reduce health disparities and improve access to services, including health care, social services, employment, and education.^2^ Service providers are often the first point of contact for resettled refugees and play a critical role in helping them to adjust to life in a new country.^3^ However, cultural competence approaches in refugee service settings continue to be limited by a lack of clear definitions and operational guidance, and insufficient attention to the unique challenges faced by people from refugee backgrounds.^1,4–6^

There is considerable variation and inconsistency in the definition of cultural competence.^7,8^ One frequently cited definition describes cultural competence as:

a set of congruent behaviors, attitudes, and policies that come together in a system, agency or among professionals and enables that system, agency or those professionals to work effectively in cross-cultural situations.^9^^(p13)^

Cultural competence has also been defined in health care settings as:

the ability of systems to provide care to patients with diverse values, beliefs and behaviors, including tailoring delivery to meet patients' social, cultural, and linguistic needs.^10^^(p5)^

Furthermore, Sue et al.^11^ described three attributes of culturally competent service providers—cultural awareness, cultural knowledge, and cultural skills—which have been replicated in several cultural competence models.^8^

The notion of cultural competence, as expressed in service settings, has been critiqued in the literature.^1,6^ Hwang et al., for example, noted that “professionals who want and need to be culturally competent are left with the message that culture matters, but continue to struggle with how to be a more culturally competent practitioner in concrete terms.”^5^ Compounding the lack of conceptual clarity and operational guidance,^1,6^ cultural competence approaches have also been criticized for overemphasizing cultural traits and differences; conflating culture with ethnicity, nationality, or language; and reducing complex human behavior and experience to cultural stereotypes.^1,6^ Furthermore, the term “competence” is increasingly being rejected, as it implies a technical endpoint or solution, rather than an enduring process and commitment. Alternative concepts have emerged, including cultural safety, which draws attention to power dynamics, institutional discrimination, and issues of colonialism and paternalism in health care.^12^ A related construct, cultural humility, requires three commitments from practitioners: (1) self-reflection and critique; (2) action to redress power imbalances; and (3) partnerships with advocates.^13^ Ethnographic approaches highlight what is most important to individuals, as culturally situated subjects, without essentializing culture or assuming it is the most critical factor in a given case.^6,14^

Cultural competence has been studied most extensively in the health field, where interventions have been demonstrated to improve health care providers' knowledge, understanding, and skills when caring for patients from multicultural backgrounds.^15–19^ However, the evidence for improved clinical outcomes and health disparities is weak.^8,19^ A 2015 systematic review found a lack of evidence of impact of cultural competence on outcomes including health status, treatment adherence, equity, and quality of services.^8^

While attention to cultural competence has expanded to consider the needs of increasingly diverse populations,^1^ there is limited research that specifically examines the cultural competence of services for refugee and asylum seeker populations. We identified no published reviews that synthesize the literature on this subject and recognize refugees as a distinct population with unique needs that may differ from other immigrant populations.^20^ Despite their diverse cultural backgrounds and nationalities, refugees and asylum seekers often share common experiences, including trauma, torture, the loss or separation of family members, the hardships of flight, as well as stigma, discrimination, social isolation, financial insecurity, and protracted asylum determination processes.^2,21–23^ Studies suggest that refugees and asylum seekers may have a greater need than the general population for certain services, including mental health services, yet they access these services at lower rates.^24,25^ Recognizing the unique backgrounds and needs of refugee populations, this scoping review synthesizes the literature on cultural competence in refugee service settings.

## Methods

This study did not require institutional review board approval. A scoping review was conducted in May 2020, guided by Arksey and O'Malley's framework.^26^ This methodology is appropriate for examining and clarifying broad research questions and synthesizing evidence across disciplines to inform practice.^26,27^ An iterative search strategy was guided by the research question:

How does the peer-reviewed literature describe cultural competence in relation to services for refugee and asylum seeker populations?

Primary search terms, as summarized in Table 1, were entered into four databases (EBSCO, ProQuest, Scopus, and Google Scholar), without date limits or restrictions based on geographic setting. The search strategy and review of titles and abstracts yielded 73 original records that were screened further for eligibility. Of these, 55 articles were selected for full-text review; 2 articles were excluded as only abstracts were available. A total of 26 articles met the inclusion criteria (Table 2). Eligible literature included peer-reviewed articles published in English that examined cultural competence as it related to refugee or asylum seeker populations. Gray literature and records that did not explicitly include refugees or asylum seekers were excluded.

**Table 1.** Primary Search Terms

“refugee” OR “asylum seeker” OR “asylee”	AND
“cultural competence” OR “cultural competency” OR “culturally competent” OR “cross-cultural” OR “culturally appropriate” OR “culturally sensitive”	AND

**Table 2.** Inclusion and Exclusion Criteria

Criteria	Criteria	Exclusion
Language	English	Language other than English
Publication type	Peer-reviewed article	Any publication type not mentioned in the inclusion criteria, for example, book chapter, letter, conference abstract
	Review	
Population	Refugees	Populations not mentioned in the inclusion criteria, including U.S. citizens and permanent residents and other documented or undocumented immigrants
	Asylees	
	Asylum seekers	
Study types	Qualitative research	Studies not mentioned in the inclusion criteria
	Quantitative research	
	Mixed methods research	
	Review	
	Case study	
	Theoretical model	
	Discussion paper	

All articles that met the inclusion criteria were uploaded and analyzed by the primary reviewer using Dedoose Qualitative Software (2018). Information on study characteristics was extracted and tabulated, and key qualitative themes were derived by thematic analysis. A second reviewer independently reviewed a subset of articles to validate emerging themes and study characteristics. Key themes were discussed and agreed upon by both reviewers.

## Results

Table 3 summarizes the characteristics of the 26 articles examined, including 13 qualitative studies, 7 discussion papers, 3 mixed methods studies, 2 reviews, and 1 quantitative study. Twenty-one studies considered refugees, 2 examined asylum seekers, and 3 included both groups. Among the 15 primary research studies, 8 considered service providers, 3 considered refugees or asylum seekers and 4 considered both populations. Only five studies addressed refugee perspectives directly.^21,22,28–30^ Four of these also explored provider perspectives, typically in greater numbers than refugee participants; and only one author reported validating the research findings by consulting refugee participants.^22^ The 23 articles that specified study setting were based in the United States (*n*=14), Australia (*n*=4), the Netherlands (*n*=2), England (*n*=1), Canada (*n*=1), and Scotland (*n*=1). An additional two reviews^31,32^ drew from studies conducted across several high-income countries. Of the six articles that specified the country of origin of refugee participants or service recipients, 24 countries were represented, with Iraq (*n*=4), Somalia (*n*=4), Cambodia (*n*=3), the Democratic Republic of the Congo (*n*=3), Bhutan (*n*=2), Bosnia (*n*=2), Burundi (*n*=2), Burma (*n*=2), and Sudan (*n*=2) mentioned most frequently.

**Table 3.** Characteristics of Included Studies (*n*=26)

Author and publication year	Title	Population of interest	Service setting	Study participants (if applicable)	Study approach	Study setting and refugee home countries (where stated)
1. Ballard-Kang, 2017	Using culturally appropriate, trauma-informed support to promote bicultural self-efficacy among resettled refugees: a conceptual model	Refugees	Refugee services, including health, education, and social services	N/A	Discussion paper: presents a conceptual framework for bicultural self-efficacy	USA
2. Burchill and Pevalin, 2014	Demonstrating cultural competence within health-visiting practice: working with refugee and asylum-seeking families	Refugees and asylum seekers	Health care (primary care)	*N*=14 service providers (health visitors, registered nurses with public health qualifications)	Qualitative: in-depth interviews	London, England
3. Campbell and Turpin, 2010	Refugee settlement workers' perspectives on home safety issues for people from refugee backgrounds	Refugees, newly arrived	Resettlement services (home safety visits)	*N*=16 service providers (resettlement service workers)	Qualitative: semi-structured interviews and observation	Australia
4. Dana, 1998	Cultural competence in three human service agencies	Refugees, Native American Indians and Hispanic Americans	Social services	N/A	Discussion paper	USA
5. Downs, Bernstein, and Marches, 1997	Providing culturally competent primary care for immigrant and refugee women: a Cambodian case study	Refugee and immigrant women	Health care (primary care)	*N*=1 refugee	Qualitative: case study	USA Home country: Cambodia
6. Dubus and Davis, 2018	Culturally effective practice with refugees in Community Health Centers: an exploratory study	Refugees	Health care (mental health) and social services	*N*=15 service providers (refugee behavioral health services providers including social workers, psychologists, psychiatrists and program managers)	Qualitative: semi-structured interviews	Northeast USA Providers served refugees from countries including Bhutan, Bosnia, Burundi, Burma, Cambodia, Congo, Djibouti, Eritrea, Iraq, Nepal, Somalia, and Sudan
7. George, 2012	Migration traumatic experiences and refugee distress: implications for social work practice	Refugees	Social services	N/A	Discussion paper	N/A
8. Grant, Parry, and Guerin, 2013	An investigation of culturally competent terminology in healthcare policy finds ambiguity and lack of definition	“Culturally marginalized populations,” particularly women and children from refugee backgrounds	Health care	N/A	Qualitative: document analysis	Adelaide, South Australia, Australia
9. Griswold, Zayas, Kernan, and Wagner, 2007	Cultural awareness through medical student and refugee patient encounters	Refugees	Health care	*N*=27 service providers (medical students who interacted with 30 refugee patients)	Qualitative: semi-structured interviews	Upstate New York, USA
10. Handtke, Schilgen and Mösko, 2019	Culturally competent healthcare—a scoping review of strategies implemented in healthcare organizations and a model of culturally competent healthcare provision	“Migrants and culturally and linguistically diverse patients,” including refugees and asylum seekers	Health care	N/A	Scoping review	Multiple countries, mostly high-income countries: 76% of studies were based in the USA
11. Kaczorowski, Williams, Smith, Fallah, Mendez, and Nelson-Gray, 2011	Adapting clinical services to accommodate needs of refugee populations	Refugees	Health care (mental health)	N/A	Discussion paper: presents lessons learned from authors' experience adapting mental health services for refugee populations	USA Clinic serves refugees from countries including Iraq, Bhutan, Burundi, Somalia, Morocco, Liberia, Congo, Vietnam, Cambodia, and Mexico
12. MacNamara, Wilhelm, Dy, Andiman, Landau, Poshkus, and Feller, 2014	Promoting quality care for recently resettled populations: curriculum development for internal medicine residents	Refugees, recently resettled	Health care (internal medicine)	*N*=155 service providers (internal medicine residents) comprising 147 assessments and 8 focus group participants	Mixed methods: Qualitative: focus groups, qualitative curriculum evaluation Quantitative: assessment survey	USA
13. Mollah, Antoniades, Lafeer, and Brijnath, 2018	How do mental health practitioners operationalise cultural competency in everyday practice? A qualitative analysis	Immigrants, including refugees and asylum seekers	Health care (mental health)	*N*=31 service providers (mental health providers with experience working with immigrant patients in the previous 12 months)	Qualitative: semi-structured interviews	Victoria, Australia
14. Parajuli and Horey, 2019	Barriers to and facilitators of health services utilisation by refugees in resettlement countries: an overview of systematic reviews	Refugees	Health care (health service utilization)	N/A	Systematic review: “overview of systematic reviews”	High-income countries
15. Phillips, 2009	Intercultural Knowledge and Skills in Social Service Work with Refugees	Refugees	Social services (including social work, economic assistance, and other human services)	*N*=28 service providers (social service providers); *N*=10 refugees	Qualitative (grounded theory): observation, open-ended interviews and document reviews	USA (“upper Midwestern city with a large refugee population”) Refugee home countries: Bosnia and Somalia
16. Pottie and Hostland, 2007	Health advocacy for refugees: medical student primer for competence in cultural matters and global health	Refugees, newly arrived	Health care (primary health care) and community-based health education and advocacy	*N*=9 service providers (medical students and primary health care professionals); *N*=1 refugee	Qualitative program evaluation: semi-structured interviews	Canada
17. Quickfall, 2014	Cultural competence in practice: the example of the community nursing care of asylum applicants in Scotland	Refugees, asylum seekers, and asylees	Health care (nursing care)	*N*=21 service providers (primary care providers) and *N*=39 asylum applicant clients or patients	Qualitative (ethnography): observation, individual interviews, focus group interviews	Glasgow, Scotland
18. Rader, Lee, and Ssempijja, 2010	Culturally competent mental health services for refugees: the case for a community-based treatment approach	Refugees	Health care (mental health)	N/A	Discussion paper: critical reflection of service provision and case study of a successful clinic model	Wisconsin, USA
19. Riggs, Davis, Gibbs, Block, Szwarc, CaseyDuell-Piening, and Waters, 2012	Accessing maternal and child health services in Melbourne, Australia: reflections from refugee families and service providers	Refugees	Health care (maternal and child health)	*N*=18 service providers (MCH nurses, bicultural workers and other health care providers); *N*=87 refugee mothers	Qualitative: focus groups and interviews	Melbourne, Victoria, Australia Refugee home countries: Iraq, Burma, Lebanon, South Sudan, and Bhutan
20. Rowe and Paterson, 2010	Culturally competent communication with refugees	Refugees	Health care	N/A	Discussion paper	New York, USA
21. Slobodin, Ghane and Jong, 2018	Developing a culturally sensitive mental health intervention for asylum seekers in the Netherlands: a pilot study	Asylum seekers	Health care (mental health)	*N*=28 asylum seekers, comprising 11 questionnaire respondents; 17 focus group participants	Mixed methods: Qualitative: focus groups Quantitative: questionnaire	Almere, The Netherlands
22. Stockbridge, Kabani, Gallups, and Miller, 2020	Ramadan and culturally competent care: strengthening tuberculosis protections for recently resettled Muslim refugees	Refugees, recently resettled	Health care (primary care and infectious diseases) and public health	*N*=148 refugees, reported as 55 Muslim, 93 non-Muslim	Quantitative	North Texas, USA
23. Suurmond, Seeleman, Rupp, Goosen, and Stronks, 2010	Cultural competence among nurse practitioners working with asylum seekers	Asylum seekers	Health care (nursing care)	*N*=125 service providers (nurse practitioners working with asylum seekers), comprising 89 questionnaire respondents and 36 group interview participants	Mixed methods: Qualitative: focus groups Quantitative: questionnaires	The Netherlands
24. Traver, 2005	A chaotic dance of cultural competence: a participatory oral history project with immigrants and refugees	Refugees and immigrants	Health care (social work)	Not specified	Qualitative (participatory action research): focus group interviews	Southern Maine, USA Refugee home countries: Somalia, DRC, Afghanistan, Dominican Republic, El Salvador, Guatemala, Ethiopia, Sudan, and Iran
25. Upvall and Bost, 2007	Developing cultural competence in nursing students through their experiences with a refugee population	Refugees, recently resettled	Health care (community health nursing)	*N*=5 service providers (community health nursing students)	Qualitative: focus groups and portfolio reviews	Southwestern Pennsylvania, USA
26. Vu, 1994	A culturally sensitive case-management model: the experience of Southeast Asian refugees in Washington State, USA	Refugees	Resettlement services/social services	N/A	Discussion paper: describes a case management model and quantitative evaluation	Washington State, USA

The majority of studies (23) focused on health care settings, including health care in general (*n*=6), mental health care (*n*=5), primary care (*n*=4), nursing (*n*=3), social work (*n*=2), maternal and child health (*n*=1), internal medicine (*n*=1), and infectious diseases (*n*=1). Five studies referred to social service settings, including refugee resettlement services (*n*=2), home safety services (*n*=1), and other social services (*n*=2).

The following section summarizes key findings and themes that emerged from the literature, informing both individual and organizational levels of practice.

### Individual-level themes

#### Self-awareness and respect for cultural diversity

Self-awareness and respect for cultural diversity were identified in the literature as important components of cultural competence.^2,22,23,33–35^ Refugee service providers may demonstrate self-awareness by critically evaluating their own culture, beliefs, biases, and values and how they influence interactions with refugee clients.^33^ Self-awareness may also involve assessing one's own culture, race, ethnicity, gender, and class in relation to refugee clients^23^ and recognizing power imbalances.^36^ Self-awareness can help providers to avoid making assumptions, generalizations, stereotypes, or judgments about other cultures.^2,22^ One study highlighted the importance of communicating honestly and openly with refugee clients and co-workers about cultural differences,^37^ with another^29^ arguing that “acceptance of a diverse range of health beliefs, rather than an emphasis on difference, is fundamental to the delivery of culturally competent community nursing care” for refugees. Recognizing one's limitations was another element of self-awareness described in the literature, including seeking guidance from senior colleagues or referring a client to more appropriate or specialized services.^4^

#### Knowledge of refugee cultures, home countries, histories, and experiences

Cultural knowledge, including knowledge of refugees' cultural and religious beliefs and practices, ethnic identities, and languages and dialects, was highlighted in several studies, often by refugee participants.^4,4,20,23,33,38,39^ Refugees in an upper Midwestern U.S. state emphasized that it was important for U.S. and local service providers to understand the cultural norms of their community, including gender norms and religious beliefs.^40^ Somali and Bosnian refugees in Maine encouraged social service providers to learn about their cultural contexts, home countries, and refugee experiences, and to connect on a human level.^22^

Experts and providers demonstrated appreciation of the unique experiences of refugees and asylum seekers,^39^ including knowledge of refugee experiences and journeys,^4,20,23^ social, historical, and political contexts in home countries, as well as conflicts and juridical systems.^4,23,33^ For example, nurse practitioners working with asylum seekers in the Netherlands wove their knowledge of different stages of flight into health assessments of asylum seekers: considering that bone fractures may have resulted from torture or that the stress of asylum procedures and living conditions during resettlement may impact mental health.^4^ One provider recognized that knowledge of ethnic conflict or tension was imperative to identify appropriate interpreters for refugee clients, beyond a simple language match.^4^ Several studies highlighted challenges of learning about clients' cultures and backgrounds, including time pressures and highly diverse client caseloads, with some providers developing strategies to obtain targeted knowledge relevant to the services they delivered.^4,20,30,36,39,41^ Other studies highlighted the complexity and diversity of refugee communities and the importance of testing and contextualizing understandings of cultural knowledge.^2,22,34^

#### Respectfully engaging refugee clients

Service providers considered respectful engagement of refugee clients critical for advancing cultural competence.^4,20,30,33,40,41^ Listening was identified as especially important, which also required attention to unequal power dynamics in refugee–provider relationships.^22,38^ Providers were also mindful of refugees' past traumatic or negative experiences and emphasized the importance of building trust and rapport and creating a safe environment.^4,30,35,41^ Approaches to facilitating trust included listening to refugee clients' concerns and priorities, ensuring continuity of service provision, exploring and managing clients' expectations of services, and clarifying the roles of providers.^20^ Honest discussions about ethical obligations, including the rules and limits of confidentiality, and services and systems in resettlement countries were also suggested as important for cultural competence.^20^ For example, nurse practitioners highlighted the value of explaining the health care system and its separation from the immigration system to asylum seeker patients, clarifying that they had no role in organizing entry to the Netherlands and would not share confidential health information with officials assessing immigration matters.^4^

Sensitivity to difficult topics, including torture and trauma, was recognized as a crucial aspect of cultural competence with refugees.^4,39^ For example, nurse practitioners reported prefacing certain questions with a statement, “I am going to ask some questions that may be painful”^4^ and recognized that apparently routine questions regarding a person's marital status or children may be distressing for refugees who have lost or been separated from family members. Respectfully challenging unsafe or harmful practices, including gender-based violence and female genital cutting, was identified as an important but difficult skill for refugee service providers.^29,34,39^

Services that recognize the “whole person,” including their spiritual and social needs, were highlighted as important elements of respectful engagement.^21,35,38,42^ Refugee participants who received social services in an upper Midwestern city in the United States appealed for service providers to “be human” and to engage with empathy and respect.^22^

How about if you are the one who left this beautiful country and went to another country with a new culture, new language, new everything, how would you feel? Emotionally already it's disaster inside. You are adjusting, you want to know the language, you are struggling to get yourself together. Before you do that, if you see some people mistreating you, it interrupts your mind. It's like, ‘whatever I try it's not working.’^22(p190)^

### Organization-level themes

#### Organizational commitment to diversity and cultural competence

Organizational commitment to cultural competence, particularly at leadership levels, was considered critical for enabling the cultural competence of mental health practitioners in Victoria, Australia.^33^ Strategies at the organizational level include improving organizational policies and practices based on employee and client evaluations^33^ and demonstrating commitment to staff diversity. The latter may be promoted by hiring bicultural and bilingual staff and ensuring that personnel policies, human resources practices, and staff compensation packages are fair and inclusive.^21–23,31,33,38,43,44^ Flexibility in organizational policies and procedures, lower caseloads, and sufficient staffing can also enable providers to support refugee clients' needs in a more culturally sensitive way.^22,41,42^

Cultural competence training was widely recognized as a method of promoting cultural competence among refugee service providers.^21,22,29,31,31,33,39,45^ Several cultural competence training programs for medical students, medical residents, social workers, and nursing students working with refugees were described in the literature.^28,36,45,46^ These were generally positively evaluated by provider participants; however, only one study sought the perspective of a single refugee participant.^28^

Handtke et al. reported a number of organization-wide cultural competence initiatives,^31^ including the “Sick-Kids Cultural Competence Initiative” at the Hospital for Sick Children in Canada, which trained more than 2100 hospital staff as cultural competence champions. One positive impact was the increased use of in-person and telephone interpreter services in the hospital.

#### Engaging and partnering with refugee communities

Partnerships between service organizations and refugee communities can facilitate cultural competence and provide mutual benefits to providers and refugee clients.^20,21,31,33,35,41,43,44^

Ethnic communities may have the advantage of offering more culturally appropriate support to refugees, but lack knowledge of signs and symptoms of trauma; more formal systems may have greater access to information about mental health, while struggling to offer support that is culturally congruent or appropriate.^35(p30)^

Kaczorowski et al.^20^ reported that strong partnerships between mental health clinics, schools, and refugee-serving agencies improved the cultural competence of mental health services for refugees, increased trust in and engagement with clinical services, and reduced barriers to treatment. Other mental health providers reported similar positive effects from cultivating relationships with refugee communities.^33^

Refugees can foster linkages between communities and service organizations by acting as cultural brokers.^21,31,34,38^ In Australia, refugee mentors from Karen/Burmese, Assyrian/Chaldean, and South Sudanese backgrounds worked effectively with refugee families to access early childhood services.^21^

Engaging family members and other community members (including community leaders and traditional healers) in service interventions, where appropriate and desired by refugee clients, may also improve cultural competence and acceptance of services.^21^ For example, in Chicago, a family-centered mental health intervention for Bosnian refugees with post-traumatic stress disorder engaged family members and bicultural refugee facilitators from the Bosnian community.^23^ Other studies have highlighted organizational flexibility and accommodation of the routines and rhythms of everyday life of the participating community.^40^ Finally, some sources recommended attention to power dynamics and the need to ensure greater participation of refugees in defining and operationalizing cultural competence; and planning, designing, and evaluating policies, programs, and interventions.^21,37,43^

#### Integrating clients' language and culture into services

Integrating clients' language and culture into services was a common approach used to strengthen organizational cultural competence. Professional interpreter services were the most frequently cited examples of this.^20–22,31,33,38–42,46^ There was a consensus that professional interpreter services were preferable to relying on family members, friends, or other staff members, due to issues of privacy, quality, and ethics. However, several barriers to using professional interpreters were identified, including cost and time constraints and limited availability of interpreters, particularly for rare languages.^29,39,41^ Several providers, including medical students, doctors, and social workers, highlighted the critical role of interpreters as cultural guides who improved the quality of interactions with refugee clients through triangulated discussion and constructive feedback.^20,40,46^

Some studies recommended the provision of linguistically and culturally appropriate verbal, written, and visual material across the service continuum, including during scheduling, reception, appointments, referrals, and follow-up; providers believed that this improved engagement with and retention in services.^21,31,43^ Incorporating culturally appropriate terms and concepts into services may also improve organizations' cultural competence. For instance, a culturally sensitive program for Southeast Asian refugees in Long Beach, California, used cultural brokers and integrated Southeast Asian concepts of pregnancy, birth, and health into clinical practice.^38^ The use of culturally adapted or cross-cultural assessment tools, such as the Refugee Health Screener-15 (RHS-15) for emotional distress^47^ and the Cultural Formulation Interview,^48^ may also be helpful.

#### Addressing barriers to access

Assisting refugee clients to overcome barriers to access was described as important for advancing cultural competence.^20,30^ Flexible models of service delivery were commonly described in the literature.^21,38,39,41,42,43^ For example, a refugee health nurse modified her appointment times to fit the bus schedule used by many of her refugee patients^21^; refugee health clinics in the United States and Australia offered flexible drop-in hours with interpreters available^41,21^; and a North Texas clinic reported higher treatment completion rates among Muslim refugee patients after providing after-dusk home delivery of tuberculosis medications during Ramadan.^49^ Integrating or colocating services that were commonly used by refugees, such as English lessons, employment assistance, food assistance, or primary care services^21,38,39^; providing transportation assistance^38,41,44^; offering home visits, school programs, and other community-based services^20,42^; facilitating appointments and referrals^21^; and using telemedicine and digital technologies^31^ were also identified as facilitators. While many providers endeavored to be flexible and responsive to refugees' needs, they discussed the constraints of inflexible policies, procedures, and rules imposed by their organizations.

## Discussion

This scoping review of 26 peer-reviewed articles identified a range of approaches to cultural competence in refugee service settings, generally described at individual and organizational levels. At the individual level, self-awareness and respect for cultural diversity; knowledge of refugee cultures, journeys, and experiences; and respectfully engaging with refugee clients were emphasized. At the organizational level, a commitment to cultural competence and diversity; engaging and partnering with refugee communities; integrating clients' language and culture into services; and addressing barriers to access were highlighted. Humility, flexibility, and a commitment to ongoing learning and development were unifying themes across the literature.

Refugee perspectives, although limited, emphasized the importance of providers who demonstrated respect and empathy and understood the culture and lived experience of refugees.

Several approaches were consistent with the broader cultural competence literature, including using professional interpreters, leveraging bicultural and bilingual staff and cultural brokers, cultural competence training, integrated care models, family-centered or community-based service models, and the integration of culturally specific concepts and cross-cultural assessments into service provision.^5,31^ Providers also described responding to the unique needs and experiences of refugees, paying particular attention to issues of trust and safety; histories of trauma, torture, or bereavement; political situations and ethnic conflicts in clients' home countries; and health risks and stressors at different stages of the refugee journey. Providers also emphasized the value of exploring and managing refugees' expectations of services, and explaining the roles of providers and national systems in resettlement countries.

It is notable that the literature focused largely on the United States and other high-income countries, and on health care and social service settings. A lack of conceptual clarity, methodological rigor, and comparative study designs meant that it was not possible to draw conclusions about which cultural competence approaches were most effective, or to generalize the findings to other refugee populations or service settings. Indeed, cultural competence is likely to be context-specific, given the heterogeneity of refugee populations and the services they use. As stated by Riggs:

there may not be one ‘model’ of best practice … but a suite of strategies that are flexible and adaptable and are reflective of the clients' cultures, languages, existing social groups and resources of local service providers—both mainstream and culturally- specific.^21(p14)^

The cultural competence literature in refugee service settings reflected a lack of meaningful participation of the populations intended to benefit from cultural competence. Refugee voices were conspicuously underrepresented in the studies identified. The literature in this review was primarily informed by the perspectives of experts^50^ and health care professionals, including doctors, nurses, psychologists, and social workers. The literature relied heavily on providers' self-reported understanding of their own cultural competence, and subjective perceptions of cultural competence outcomes, with little awareness of how these might be shaped by intersubjective interactions with refugee clients.

Interestingly, few articles included in this review disclosed or discussed the ethnic or cultural identity of providers, suggesting that their cultural values, norms, and practices were assumed, normalized, or perhaps considered less pertinent to the topic of cultural competence. Insufficient attention to the cultures of all parties in a client–provider relationship may hinder our understanding of cultural competence, or support narratives that present “other cultures” (typically nondominant cultures) as problematic. The notion of cultural competence is itself a culturally determined construct that is embedded in historically constituted power relations.

Anthropological approaches referred to by some authors may be broadly instructive for service providers working with refugees. Kleinman and Benson's Explanatory Models Approach and revised cultural formulation (an ethnographic approach describing six steps for culturally informed clinical practice) seek to understand “what really matters” and “what is at stake” for patients, their families, and their communities, and to use this information to guide clinical diagnoses, decision-making, and negotiations with patients.^6^ These models require providers to “set their expert knowledge alongside, not over and above the patient's own explanation and viewpoint.”^6^ Potocky-Tripodi suggested that social workers seeking to provide more culturally sensitive services to refugees should pose the question, “what would you like me to know so I can help you better?”^51^ These approaches advance beyond viewing cultural competence as a set of technical skills to acquire or procedures to deliver, instead placing refugees at the center of the services they receive.

The literature was clear that an enabling organizational environment is key for opening up the institutional space required to achieve the goals of cultural competence. This can be facilitated by championing the values of cultural competence at leadership levels, advancing staff diversity, implementing more flexible policies, procedures and service delivery models, and partnering with refugee communities. While some refugee participants described structural barriers, including stigma, discrimination, racial profiling, and fears of interacting with authorities and government services,^40^ recognition of these structural barriers was an important gap in the literature. Until structural inequalities impacting service quality and accessibility for refugees and other marginalized populations are recognized and addressed, the ideals of cultural competence will likely remain elusive.

### Limitations

The nonexhaustive search strategy and reliance on peer-reviewed literature published in English is a limitation of this review. The included literature was largely U.S.-focused, and confined to health and social service settings, particularly mental health. Relevant publications, including the gray literature and literature published in other languages, disciplines, or service settings, may not have been located. In addition, as previously noted, the lack of refugee perspectives is a significant limitation of this review.

## Conclusion

This scoping review identified a range of individual and organizational approaches to cultural competence in refugee service settings, including strategies responsive to the unique circumstances and needs of refugees. A lack of refugee perspectives and insufficient attention to structural barriers were notable gaps in this literature.

Future research on cultural competence in refugee service settings requires greater attention to what cultural competence means to people with refugee status, how they experience it, and how this is shaped by the social, political, and economic contexts in which they emerge. Otherwise, cultural competence approaches risk reproducing the same cultural hierarchies and structural inequities that they aim to address.
